# Liver Enzymes: Interaction Analysis of Smoking with Alcohol Consumption or BMI, Comparing AST and ALT to γ-GT

**DOI:** 10.1371/journal.pone.0027951

**Published:** 2011-11-22

**Authors:** Lutz P. Breitling, Volker Arndt, Christoph Drath, Hermann Brenner

**Affiliations:** 1 Division of Clinical Epidemiology and Aging Research C070, German Cancer Research Center (DKFZ), Heidelberg, Germany; 2 Workmen's Compensation Board for Construction Workers, Occupational Health Service, Böblingen, Germany; The University of Hong Kong, Hong Kong

## Abstract

**Background:**

A detrimental interaction between smoking and alcohol consumption with respect serum γ-glutamyltransferase (γ-GT) has recently been described. The underlying mechanisms remain unknown. The present work aimed to provide further insights by examining similar interactions pertaining to aspartate and alanine transaminase (AST, ALT), routine liver markers less prone to enzyme induction.

**Methodology/Principal Findings:**

The present cross-sectional analysis was based on records from routine occupational health examinations of 15,281 male employees predominantly of the construction industry, conducted from 1986 to 1992 in Southern Germany. Associations of smoking intensity with log-transformed activities of γ-GT, AST, and ALT were examined in regression models adjusted for potential confounders and including an interaction of smoking with alcohol consumption or body mass index (BMI). Statistically significant interactions of smoking were observed with both alcohol consumption (AST and ALT, each with *P*<0.0001) and BMI (AST only, *P*<0.0001). The interactions all were in the same directions as for γ-GT, i.e. synergistic with alcohol and opposite with BMI.

**Conclusion:**

The patterns of interaction between smoking and alcohol consumption or BMI with respect to AST and ALT resembled those observed for γ-GT. This renders enzyme induction a less probable mechanism for these associations, whereas it might implicate exacerbated hepatocellular vulnerability and injury.

## Introduction

Routine markers of liver function and damage [1], [2], in particular γ-glutamyltransferase (γ-GT), aspartate and alanine transferase (AST and ALT, respectively), have attracted substantial interest in recent years because numerous studies suggested a predictive potential regarding a variety of clinical outcomes in both generally healthy and in patient cohorts [3]–[12]. Knowledge of causal relationships linking liver enzymes with clinical outcomes remains very limited, and better understanding the role of disease risk factors in the inter-individual variation of liver enzyme activities would be of high interest.

In a recent study, we found a detrimental synergistic interaction between smoking and alcohol consumption with respect to the elevation of serum γ-GT [13]. Such an interaction would apply to a large number of individuals due to the high frequency and co-occurrence of tobacco and alcohol use and abuse. Intriguingly, we subsequently obtained evidence that the interaction between smoking and body mass index (BMI), another important determinant of serum γ-GT, might be opposite to the smoking×alcohol interaction, i.e. smoking intensity was positively associated with γ-GT only in subjects with low rather than high BMI, although BMI itself—just like alcohol consumption—is positively associated with higher serum γ-GT [14].

In the present work, we extended our considerations to AST and ALT. If the interaction effects previously described for γ-GT were primarily due to enzyme induction, a mechanism known to apply predominantly to γ-GT [15], they should not or not to the same extent become apparent in analyses of AST and ALT. Serum activities of both these enzymes, however, respond well to compromised liver cell integrity [1]. Thus, the presence of similar interactions as observed for γ-GT would suggest that an escalation of tissue damage in the central homeostatic organ plays a relevant role in the observed associations.

## Methods

### Ethics Statement

Whereas participation in the occupational health exams forming the basis of this work is non-mandatory for most occupation groups according to German occupational safety laws, anonymised data obtained in such exams is to be collected and analysed scientifically. Thus, no additional specific informed consent was required for analysis of anonymised data in this project. However, the study protocol was approved by the ethics boards of Heidelberg University, Ulm University, and by the Ministry of Social Affairs of the State of Baden-Württemberg. As the ethics boards generally do not express approval of each individual aspect of the study, but rather—if necessary—request individual aspects of the study to be improved, the need for informed consent was not explicitly addressed in their approval of the study protocol.

### Design, Setting, and Participants

Data used in the present study originated from routine occupational health examinations (April 1986 to December 1992), for which records were obtained from the Workmen's Compensation Board for Construction Workers in Württemberg in South Germany. Details of the study design have been reported previously [4], [11], [14]. Only males were included, and the study population is representative for a large number of construction workers in Germany.

### Data Collection

The health examinations were conducted by experienced occupational health professionals in a standardised manner. They included taking a blood sample for routine laboratory analysis. In addition, participants were asked to fill out a standardised questionnaire covering occupational information, nationality, health behaviour (smoking, alcohol consumption), prevalent disease (coded according to ICD-9), and body weight and height. Serum activities of liver enzymes were determined centrally as part of the routine exam, using a Hitachi 705/717 instrument working at 25°C, with upper reference limits of 28, 18, and 22 U/L for γ-GT, AST, and ALT, respectively (corresponding to approx. 49, 38, and 41 U/L when converted to 37°C [16]).

### Statistical Analysis

The study population was first described regarding the distribution of important potential confounding characteristics, including the medians (interquartile ranges) and frequencies of above normal activities of the three enzymes within each covariable stratum. The statistical significance of differences between the strata was assessed by Kruskal-Wallis testing.

Interactions between smoking intensity and alcohol consumption or BMI on liver enzyme activities were examined using linear regression models adjusted for age (<25, 25–34, 35–44, 45–54, ≥55 years), alcohol consumption (none, occasional, 1–30, 31–60, 61–90, >90 g/day), BMI (<25, 25–<30, ≥30 kg/m^2^), nationality (German, Italian, Turkish, Yugoslavian, other), professional group (bricklayer, carpenter, office worker, painter, plasterer, plumber, unskilled worker), diabetes (ICD-9 250), hypertension (ICD-9 401-405), and ischemic heart disease (ICD-9 410-414). For these regression models, former smokers and consumers of tobacco products other than cigarettes were excluded (these subjects form a subgroup that must be considered highly heterogeneous and do not readily fit into an analysis of current smoking exposure), and smoking was coded as a trend variable taking on the median smoking intensity in cigarettes per day (cpd) within each smoking stratum (these medians were 0, 10, 20, and 30 cpd, in the categories of never smokers, smokers of <20, 20, and >20 cpd, respectively). The interaction was judged based on the significance of the interaction term between the smoking trend variable and the alcohol consumption or BMI stratum variable (tests with 5 and 2 degrees of freedom, respectively).

In the linear regression models, liver enzymes were natural log-transformed throughout, because all three showed a right-skewed distribution. For sensitivity analyses, subjects with any of the liver enzyme activities, BMI, alcohol consumption or smoking intensity beyond the 99^th^ or 95^th^ percentile were excluded. We furthermore explored the impact of estimating the smoking trend association in models fitted separately to each BMI or alcohol stratum while including age, age^2^, BMI, BMI^2^, and alcohol consumption as continuous variables. All statistical analyses were conducted using SAS 9.2 (SAS Institute, Cary/NC), using two-sided tests and α = 5%.

## Results

### Descriptive Analysis

A total of 22,014 health exam records included measurements of all three liver markers. After excluding those individuals lacking data on smoking (or smoking tobacco products other than cigarettes), alcohol consumption, or BMI, 15,281 subjects remained in our analysis set. This was a slightly different analysis population than in the in-depth report focussed entirely on γ-GT, which explains minimal differences of the present compared to the previous γ-GT results [14].

Median activities of the markers by covariable categories are reported in **Table 1**. Distributions of all three markers were right-skewed, the 95th and 99th percentiles being 96 and 248, 25 and 51, and 38 and 66 for γ-GT, AST and ALT, respectively. The markers were strongly correlated with Spearman coefficients of 0.52 (γ-GT, AST), 0.63 (γ-GT, ALT), and 0.70 (AST, ALT), which remained essentially the same when controlling for age.

**Table 1:** Median and interquartile range (IQR) of serum γ-GT, AST and ALT (at 25°C) in n = 15281 working age males in Southern Germany, according to sociodemographics, occupation, nationality, life-style factors and prevalent diseases.

Characteristic		n	%	Serum γ-GT (U/L)				Serum AST (U/L)				Serum ALT (U/L)			
				Median	(IQR)	>28 U/L (%)	*P* a	Median	(IQR)	>18 U/L (%)	*P* a	Median	(IQR)	>22 U/L (%)	*P* a
Total population		15281	100.0	17.0	(12.0–31.0)	28.3	na	11.0	(9.0–14.0)	10.8	na	14.0	(11.0–20.0)	20.0	na
Age	<25 years	1598	10.5	12.0	(9.0–17.0)	8.9		10.0	(9.0–12.0)	5.7		11.0	(9.0–15.0)	9.3	
	25–34 years	4339	28.4	15.0	(11.0–26.0)	22.4		11.0	(9.0–14.0)	10.1		14.0	(11.0–21.0)	21.2	
	35–44 years	2904	19.0	20.0	(13.0–38.0)	35.0		11.0	(9.0–14.0)	13.0		16.0	(12.0–23.0)	26.2	
	45–54 years	4333	28.4	20.0	(13.0–38.0)	35.2		11.0	(9.0–14.0)	12.4		15.0	(11.0–21.0)	20.8	
	≥55 years	2107	13.8	20.0	(13.0–34.0)	32.0	<0.0001	11.0	(9.0–13.0)	9.7	<0.0001	14.0	(10.0–19.0)	15.6	<0.0001
Nationality	German	11595	76.1	18.0	(12.0–33.0)	30.1		11.0	(9.0–14.0)	11.1		14.0	(11.0–20.0)	20.1	
	Italian	1074	7.0	17.0	(12.0–29.0)	25.3		11.0	(9.0–14.0)	10.1		15.0	(11.0–21.0)	21.3	
	Turkish	814	5.3	12.0	(9.0–17.0)	7.0		10.0	(8.0–12.0)	3.3		12.0	(9.0–17.0)	10.7	
	Yugoslavian	1295	8.5	18.0	(11.0–32.0)	28.6		11.0	(9.0–15.0)	13.1		15.0	(11.0–22.0)	24.0	
	Other	459	3.0	16.0	(11.0–30.0)	26.4	<0.0001	11.0	(9.0–13.0)	10.0	<0.0001	14.0	(11.0–20.0)	17.9	<0.0001
Occupational group	Bricklayer	4714	30.8	19.0	(12.0–34.0)	31.2		11.0	(9.0–14.0)	11.7		15.0	(11.0–21.0)	21.2	
	Carpenter	2070	13.5	16.0	(11.0–28.0)	24.8		11.0	(9.0–14.0)	8.8		14.0	(11.0–20.0)	17.7	
	Painter	2223	14.5	17.0	(12.0–32.0)	28.5		11.0	(9.0–13.0)	9.9		14.0	(10.0–20.0)	19.9	
	Plasterer	1560	10.2	18.0	(11.0–33.0)	30.1		11.0	(9.0–14.0)	11.2		14.0	(10.0–21.0)	20.9	
	Plumber	2346	15.4	17.0	(11.0–29.0)	26.0		11.0	(9.0–13.0)	9.4		14.0	(11.0–20.0)	19.1	
	Unskilled	2167	14.2	17.0	(11.0–30.0)	27.2		11.0	(9.0–14.0)	13.4		14.0	(11.0–20.0)	20.4	
	Office	201	1.3	15.0	(11.0–26.0)	19.9	<0.0001	10.0	(9.0–12.0)	6.0	<0.0001	13.0	(9.0–19.0)	14.9	0.0004
Cigarette smoking	Never	4121	27.0	16.0	(11.0–27.0)	22.7		11.0	(9.0–13.0)	8.2		14.0	(11.0–20.0)	18.7	
	<20 cpd	2923	19.1	16.0	(11.0–29.0)	25.1		11.0	(9.0–13.0)	9.8		14.0	(10.0–20.0)	18.6	
	20 cpd	3689	24.1	17.0	(12.0–32.0)	28.5		11.0	(9.0–14.0)	11.2		14.0	(10.0–19.0)	17.4	
	>20 cpd	1922	12.6	20.0	(13.0–40.0)	36.2		11.0	(9.0–15.0)	15.8		15.0	(11.0–21.0)	22.5	
	Formerly	2626	17.2	20.0	(13.0–36.0)	34.5	<0.0001	11.0	(9.0–14.0)	11.6	<0.0001	16.0	(12.0–23.0)	25.4	<0.0001
Alcohol consumption	None	1762	11.5	12.0	(9.0–18.0)	8.7		10.0	(8.0–12.0)	4.1		13.0	(10.0–17.0)	11.9	
	Occasionally	6062	39.7	15.0	(10.0–24.0)	18.2		10.0	(9.0–13.0)	5.5		13.0	(10.0–19.0)	15.7	
	1–30 g/d	1569	10.3	16.0	(11.0–27.0)	22.4		10.0	(9.0–13.0)	6.9		14.0	(10.0–19.0)	17.3	
	31–60 g/d	2915	19.1	21.0	(13.0–38.0)	36.1		11.0	(9.0–14.0)	11.7		15.0	(11.0–21.0)	22.2	
	61–90 g/d	1455	9.5	28.0	(16.0–52.0)	49.8		13.0	(10.0–17.0)	20.0		16.0	(12.0–24.0)	28.5	
	>90 g/d	1518	9.9	38.0	(21.0–74.0)	61.9	<0.0001	14.0	(11.0–21.0)	32.9	<0.0001	18.0	(13.0–28.0)	37.1	<0.0001
Body Mass Index	<25 kg/m^2^	6232	40.8	13.0	(10.0–22.0)	17.8		11.0	(9.0–13.0)	10.3		12.0	(9.0–16.0)	11.3	
	25–<30 kg/m^2^	6899	45.1	20.0	(13.0–34.0)	32.3		11.0	(9.0–14.0)	10.3		15.0	(12.0–21.0)	22.2	
	≥30 kg/m^2^	2150	14.1	26.5	(17.0–46.0)	46.2	<0.0001	12.0	(10.0–15.0)	13.9	<0.0001	19.0	(14.0–27.0)	38.1	<0.0001
Prevalent diabetes	no	14617	95.7	17.0	(11.0–31.0)	27.4		11.0	(9.0–14.0)	10.5		14.0	(10.0–20.0)	19.5	
	yes	664	4.3	28.0	(17.0–55.0)	48.6	<0.0001	11.0	(9.0–15.0)	17.0	0.0025	17.0	(12.0–25.0)	31.3	<0.0001
Prevalent ischemic heart disease	no	15057	98.5	17.0	(11.0–31.0)	28.2		11.0	(9.0–14.0)	10.8		14.0	(11.0–20.0)	20.0	
	yes	224	1.5	24.0	(14.5–41.0)	37.9	<0.0001	11.0	(9.0–14.0)	8.5	0.51	15.0	(12.0–21.0)	21.9	0.029
Prevalent hypertension	no	12166	79.6	16.0	(11.0–27.0)	23.5		11.0	(9.0–13.0)	9.0		14.0	(10.0–19.0)	17.1	
	yes	3115	20.4	27.0	(16.0–51.0)	47.1	<0.0001	12.0	(10.0–16.0)	17.7	<0.0001	17.0	(12.0–25.0)	31.2	<0.0001

a *P*-value of Kruskal-Wallis test.

Above-normal levels were most prevalent for γ-GT, and least for AST. Associations of the three markers were apparent with most major participant characteristics, and were sometimes very pronounced (Table 1). Both AST and ALT appeared positively associated with alcohol consumption and BMI.

### Interaction Analyses

**Figure 1:**
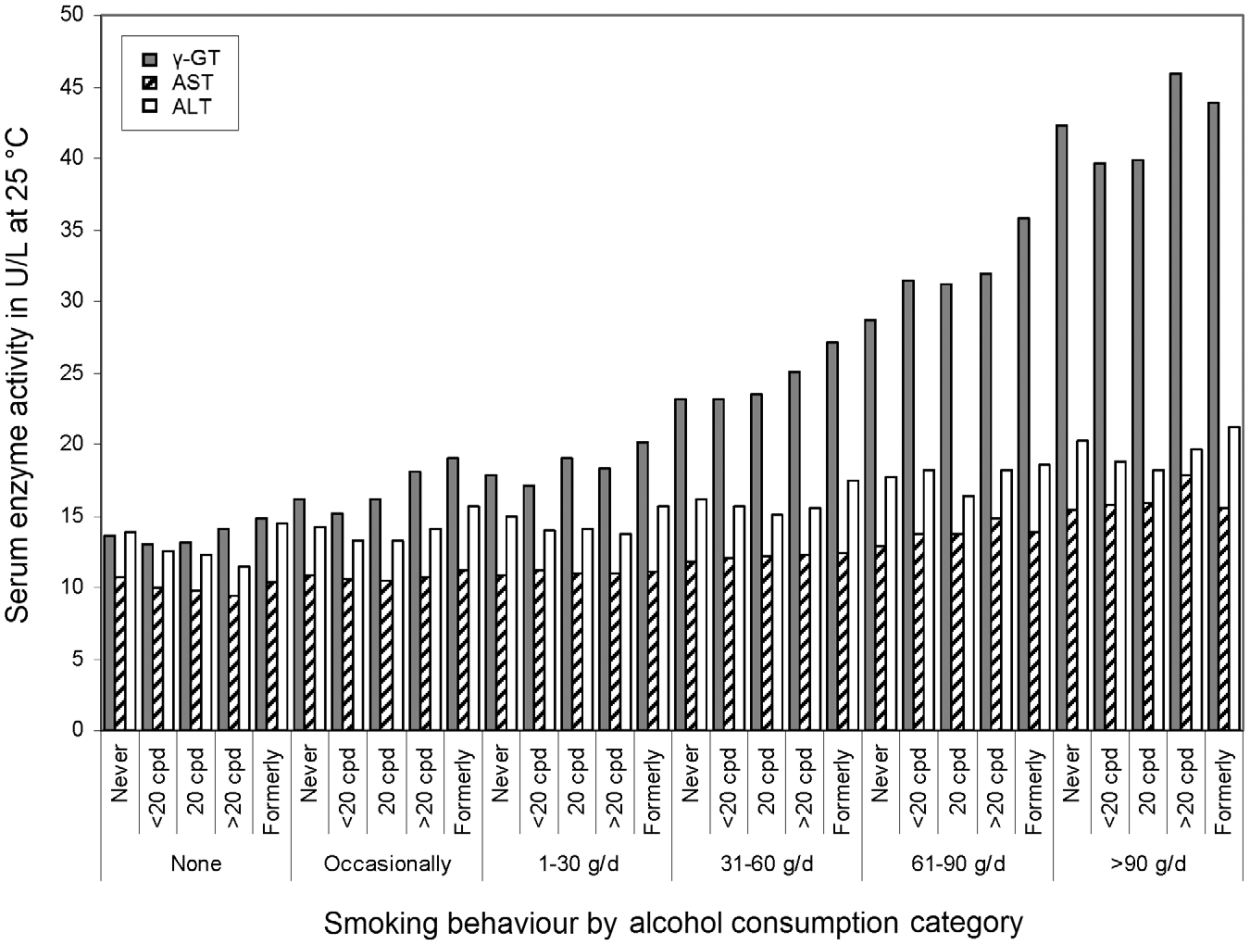
Geometric mean serum activities of γ-GT (grey bars), AST (hatched bars) and ALT (white bars; all in U/L measured at 25°C) in 15,281 men in Germany, by alcohol consumption intensity (bottom row) and smoking behaviour (cpd = cigarettes per day).

Geometric mean activities of the liver enzymes by smoking×alcohol drinking strata are shown in **Figure 1** (for cell sizes, medians and interquartile ranges of the activities, see **Table S1**). An increase across rising alcohol consumption intensity was apparent for all three enzymes, but smoking effects appeared less consistent in these crude analyses. However, there seemed to be some tendency of smoking intensity to be associated negatively with AST and ALT activities in subjects not drinking alcohol and positively or with inconsistent pattern in subjects with higher alcohol consumption. Former smokers tended to show the highest concentration of each marker within most drinking categories.

**Figure 2:**
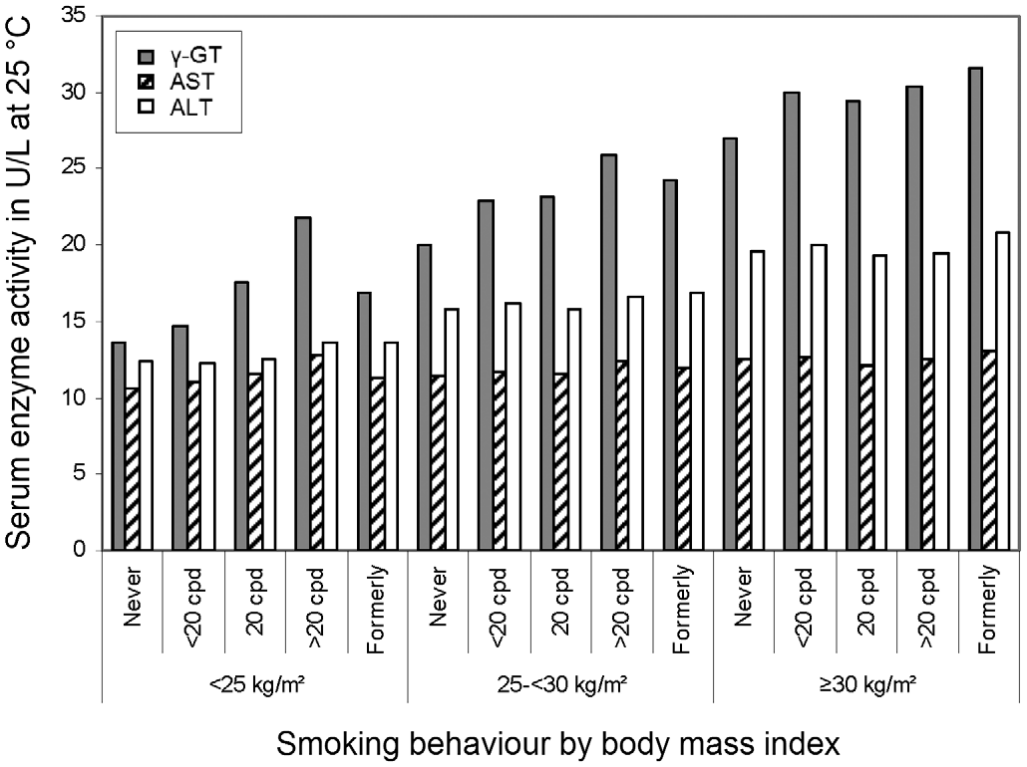
Geometric mean serum activities of γ-GT (grey bars), AST (hatched bars) and ALT (white bars; all in U/L measured at 25°C) in 15,281 men in Germany, by body mass index and smoking behaviour (cpd = cigarettes per day).

The interaction of smoking×BMI regarding geometric mean activities of the three markers is depicted in **Figure 2** (for cell sizes, medians and interquartile ranges of the activities, see **Table S2**). For γ-GT and ALT, there was a clear positive association with BMI. In subjects with BMI <25 kg/m^2^, a strong positive association with smoking intensity was seen for γ-GT, whereas the association with AST was much less pronounced and the one with ALT hardly visible. In obese participants, γ-GT was lowest in never smokers, but otherwise there was little evidence for an association between the markers and smoking.

In linear regression models predicting the log-transformed marker activities from smoking×alcohol consumption intensity or smoking×BMI strata, adjusted for age group and BMI (in case of the smoking×alcohol interaction) or alcohol drinking intensity (in case of the smoking×BMI interaction), the interaction tendencies described in the preceding paragraph became clearer (not shown). Compared to never-smokers with BMI<25 kg/m^2^, AST and ALT appeared to be less elevated in heavily smoking obese than in never-smoking obese subjects. The estimates hardly changed when additionally adjusting for the remaining covariables listed in Table 1.

**Table 2** presents the fully adjusted main models of the present analyses, in which the association of smoking intensity coded as a trend variable (see Methods section) with each serum marker was evaluated along with its interaction with alcohol consumption or BMI. These models suggested pronounced and mostly statistically significant interaction effects for AST and ALT. The interactions were directionally similar to those described for γ-GT. However, whereas smoking showed no main effect on γ-GT in alcohol abstinent and obese subjects, and a positive association in heavy drinkers or subjects with normal weight, the patterns were somewhat shifted for AST and ALT, for which there was a negative association of smoking intensity with the respective marker in alcohol abstinent and obese subjects, and a positive (AST) or no association (ALT) in heavy drinkers or normal weight subjects. When both interaction terms were included together in the models, the individual *P*-values hardly changed (not shown).

**Table 2:** Fully adjusted association (see footnote) of smoking intensity with liver enzymes by drinking and body mass index strata, estimated in linear regression models predicting natural log-transformed enzyme activities.

Interaction stratum		Change in γ-GTper 10 cpd				Change in ASTper 10 cpd				Change in ALTper 10 cpd			
		Δ%	(95% CI)		*P* a	D%	(95% CI)		*P* a	Δ%	(95% CI)		*P* a
Alcohol consumption	None	0.76	(−2.39	to 4.01)		−4.45	(−6.14	to −2.73)		−6.00	(−8.07	to −3.88)	
	Occasional	4.35	(2.49	to 6.25)		−0.93	(−1.93	to 0.08)		−1.77	(−3.00	to −0.52)	
	1–30 g/day	5.49	(1.74	to 9.37)		0.11	(−1.90	to 2.16)		−2.33	(−4.77	to 0.18)	
	31–60 g/day	5.90	(3.14	to 8.74)		1.48	(−0.02	to 2.99)		−0.95	(−2.77	to 0.90)	
	61–90 g/day	8.41	(4.60	to 12.4)		4.17	(2.10	to 6.29)		1.29	(−1.22	to 3.87)	
	>90 g/day	7.76	(4.02	to 11.6)	0.028	5.30	(3.23	to 7.40)	<0.0001	1.50	(−0.98	to 4.04)	<0.0001
Body mass index	<25 kg/m^2^	7.24	(5.36	to 9.15)		2.05	1.05	to 3.07)		−0.67	(−1.89	to 0.57)	
	25–<30 kg/m^2^	3.98	(2.25	to 5.74)		−0.58	−1.51	to 0.37)		−2.23	(−3.37	to −1.07)	
	≥30 kg/m^2^	1.95	(−1.03	to 5.03)	0.0045	−2.24	−3.87	to −0.59)	<0.0001	−2.25	(−4.27	to −0.19)	0.14

a P-value of interaction test.

Note. All six models were adjusted for age, nationality, occupational group, alcohol consumption, body mass index, diabetes, ischemic heart disease, and hypertension (n = 12,615; former smokers and subjects with missing nationality were excluded).

### Sensitivity Analyses

When subjects with smoking intensity, alcohol consumption intensity, BMI, γ-GT, AST, or ALT beyond the 99th percentile (50 cpd, 200 g/day, 36.5 kg/m^2^, 248 U/L, 51 U/L, 66 U/L) were excluded for the purpose of sensitivity analyses, the Table 2 results hardly changed (details not shown). The same was true for using a the 95th percentile as cutoff (40 cpd, 125 g/day, 32.5 kg/m^2^, 96 U/L, 25 U/L, 38 U/L). Whereas the interactions with respect to γ-GT lost statistical significance in the latter models limited to 10,694 subjects, the estimated associations and interaction patterns generally remained similar in these analyses. The smoking trend estimates obtained in models stratified on BMI or alcohol consumption category and treating certain covariables as continuous and potentially non-linear predictors as detailed above were overall in line with the main analyses' results (not shown).

## Discussion

In this large cross-sectional study, interaction effects of smoking with both alcohol consumption intensity and BMI were observed with respect to serum activities of AST and ALT. Advancing our understanding of the relationships between classical disease risk factors and liver enzyme levels is important, because especially γ-GT is more and more seen as a cardiovascular risk marker, The pattern of the heterogeneity of AST and ALT associations was directionally similar to the interactions recently described for γ-GT. As alterations in AST and ALT are unlikely to be caused by enzyme induction, these observations are more in line with the hypothesis that the varying associations of smoking with liver enzymes across alcohol or BMI strata could be due to varying levels of hepatocellular vulnerability and injury.

The crude associations of the main exposures in our study were similar to previous reports. This pertains in particular to the positive associations of alcohol consumption intensity and BMI with the liver enzymes, for which the literature appears fairly consistent [17]–[22]. Some, but not all [21], previous studies suggested a stronger association of alcohol with liver enzymes in subjects with higher BMI [20], [23]. The associations with prevalent diseases already were described and discussed in the context of an earlier analysis of a subcohort of the present study [4]. An excess in particular of γ-GT in former smokers has also been described previously [13], [24]. This could be due to weight gain after cessation, but also could reflect that these subjects have quit smoking e.g. due to particular health issues that might be associated with liver enzyme elevations.

Previous studies regarding the main effect of smoking have yielded mixed results, e.g. suggesting a positive association only in women [19], or finding an effect only on γ-GT but neither on AST nor ALT [17], [18]. Such inconsistent results, of course, would not be overly unexpected if taking our main findings into account, which suggested pronounced effect heterogeneity of the association of smoking with liver enzymes to occur depending on alcohol consumption and BMI. Investigations of potential interactions between smoking and alcohol drinking with respect to serum γ-GT are very scarce [13], [24], [25]. Corresponding interaction analyses regarding AST and ALT also appear to be limited to one study [24].

In one previous investigation on smoking×alcohol interactions and serum transaminases [24], there also was a significant inverse association of smoking intensity with AST only in subjects with no or low alcohol consumption (which could mean that effects of smoking on this marker are essentially overruled by the stronger effects of alcohol consumption), but no association was found between smoking and ALT; BMI was controlled for, but its interaction with smoking was not investigated in that particular or indeed any study that has come to our attention. The authors of this previous study hypothesized that their finding of a smoking-associated elevation only in γ-GT might have been due to nicotine inducing γ-GT. In the other study of interest, the γ-GT interaction also was found, as was an inverse association of smoking with AST [25]. In contrast to the latter, the association of smoking with γ-GT was substantially reduced when adjusting for the inflammatory marker C-reactive protein, which—contrary to the previously cited study—would suggest that smoking effects on γ-GT mainly reflect inflammatory oxidative stress with an exacerbation of similar effects of alcohol in the situation of higher intensity co-consumption [13], [25].

The associations discovered in our data should allow some further insights in this regard, as pronounced and heterogeneous associations were observed between smoking and all three enzymes examined. The very robust patterns of interaction over strata of alcohol consumption or BMI with respect to AST and ALT paralleled those for γ-GT, suggesting that enzyme induction applying selectively to γ-GT [26], [27] would be at least insufficient to explain the observed associations. Interestingly, in subjects with alcoholic fatty liver, serum AST and ALT may rise in the absence of alterations at the hepatic level [15]. The interactions in similar directions and extents in comparison to γ-GT might suggest that liver injury and loss of hepatocellular integrity—potentially affecting serum activities of all three enzymes alike—should be considered as a pathophysiological mechanisms involved in creating these interaction patterns.

Whereas the arguments above would fit the explanation previously put forward regarding the smoking×alcohol interaction with respect to γ-GT, namely excessive combined oxidative stress resulting from co-consumption [13], it appears more difficult to explain the opposite interaction pattern for smoking×BMI. We earlier considered dilution effects of a higher body volume as a possible mechanism for weaker associations in obese. As obesity itself is associated with increased oxidative stress, oxidative stress would appear an unlikely explanation for the opposite directionality of effect modification across BMI in comparison to alcohol strata [28]. Interestingly, however, a recent study found smoking to be associated with a higher risk for lung cancer in subjects with lower BMI [29], and the authors cited reports of inverse correlations between BMI and markers of oxidative DNA damage to support the results' biological plausibility. Arguing along these lines, the interaction patterns observed in the present study—parallel across markers and opposite with respect to alcohol and BMI—would be in line with smoking-related serum γ-GT elevations being at least partially due to liver injury rather than enzyme induction, and excess oxidative stress could be the underlying cause of this loss of hepatocellular integrity, exacerbated—be it in absolute terms or relative to some potentially altered hepatocellular vulnerability threshold—either by high alcohol consumption or by low body weight. The previous report [25] describing that the association between smoking and AST is not affected by adjustment for C-reactive protein would suggest that the mechanisms involved in the loss of hepatocellular integrity go beyond inflammatory processes readily assessed by this marker.

A number of limitations should be considered when judging the present study's results. Although exposure assessment was conducted in a highly standardized way, smoking and drinking behaviour were determined exclusively by self-report. It appears, however, not likely that misreporting compromised the validity of our findings, as only peculiar patterns of pronouncedly differential misreporting according to γ-GT, alcohol consumption and BMI could theoretically create such complex association and interaction patterns as observed. We adjusted for a variety of important confounders, including continuous and non-linear effects in sensitivity analyses, yet we cannot rule out some residual confounding due to unknown or unmeasured factors. Physical activity would be such a potentially interesting variable, but the fairly homogenous nature of the study population rendered a serious distortion due to this or other confounders rather unlikely. Note, however, that the potential for confounding is limited, if a variable is only associated with enzyme activities, but is not a strong causal factor of the exposures of interest. For example, an additional adjustment of our models for presence or absence of regular medication—which can be considered a causal factor for liver enzyme elevations—had no impact on any of the estimates in Table 2 (data not shown). Underlying liver disease such as viral hepatitis, primary biliary cirrhosis, or autoimmune hepatitis was reported only in a very small number (<0.3%) of subjects in our cohort of workers employed in physically intensive professions, and thus was not considered in the analyses. The role of liver fat accumulation for the associations described in the present study could possibly be better understood by using imaging techniques such as ultrasound. This was not feasible in the present study setting, and only serum cholesterol, triglycerides, and fasting glucose were available as general metabolic indicators. Additional adjustment of our main regression analyses for these markers did not relevantly change the results (data not shown). Finally, longitudinal repeat measurements of the parameters analysed would have considerably strengthened attempts to draw causal conclusions, but were beyond the scope of this study. Finally, additional blood sampling for the determination e.g. of C-reactive protein was not possible in our study design.

In conclusion, the comparative analysis of both enzymes (γ-GT vs. AST and ALT) and interaction exposures (alcohol consumption vs. BMI) in the present study allowed us to suggest some pathophysiological explanations for the previously described interactions of classical risk factors as determinants of serum γ-GT. The findings tentatively implicated hepatic injury as a main mechanism involved in the detrimental smoking×alcohol interaction with respect to γ-GT. However, given the scarcity of prior investigations into potential interactions between smoking and alcohol consumption or BMI, efforts to replicate the patterns described here in independent epidemiological cohorts should be undertaken. Furthermore, especially the intriguing interactions and negative associations of smoking with AST and ALT deserve attention also from molecular and laboratory scientists. In the context of future clinical and nutritional approaches, the role of liver fat contents for these associations and interactions would be a highly interesting, though resource-demanding [30] research topic, with great potential to advance our causal understanding of these classical, yet newly discovered serum markers of liver function and integrity.

## Supporting Information

Table S1 Liver enzyme activities according to smoking * alcohol strata.
(DOC)

Table S2 Liver enzyme activities according to smoking * body mass index strata.
(DOC)
