# Supplementary material for: Liver Enzymes: Interaction Analysis of Smoking with Alcohol Consumption or BMI, Comparing AST and ALT to γ-GT
Source: PLoS One. 2011 Nov 22;6(11):e27951. doi: 10.1371/journal.pone.0027951 (PMC3222662; doi:10.1371/journal.pone.0027951)
Supplement: Table S1 — Liver enzyme activities according to smoking * alcohol strata. (DOC) [file pone.0027951.s001.doc]

| **Table S1.**  Median activities (interquartile range) of liver enzymes according to smoking * alcohol strata. | | | | | | | | | | | | | | | | | | | | |  | |  | |  | |  | |  | | |  |  | | |  |  |  | | |
| --- | --- | --- | --- | --- | --- | --- | --- | --- | --- | --- | --- | --- | --- | --- | --- | --- | --- | --- | --- | --- | --- | --- | --- | --- | --- | --- | --- | --- | --- | --- | --- | --- | --- | --- | --- | --- | --- | --- | --- | --- |
|  |  |  |  |  |  |  |  |  |  |  |  |  |  |  |  |  |  |  |  |  | |  | |  | |  | |  | |  |  | | | |  | | | |  |  |
|  |  |  |  |  |  |  |  |  |  |  |  |  |  |  |  |  |  |  |  |  | |  | |  | |  | |  | |  |  | | | |  | | | |  |  |
| **smoking** | **no alcohol consumption** | | | |  | **occasional consumption** | | | |  | **1-30 g/d alcohol** | | |  |  | **31-60 g/d alcohol** | | | |  | | **61-90 g/d alcohol** | | | | | | | |  | **91+ g/d alcohol** | | | | | | | |  | |
|  | median | IQR | | n |  | median | IQR | | n |  | median | IQR | | n |  | median | IQR | | n |  | | median | | IQR | | | | n | |  | median | | | IQR | | | | | n | |
|  |  |  |  |  |  |  |  |  |  |  |  |  |  |  |  |  |  |  |  |  | |  | |  | |  | |  | |  |  | | |  | | |  | |  | |
| **γ-GT** |  |  |  |  |  |  |  |  |  |  |  |  |  |  |  |  |  |  |  |  | |  | |  | |  | |  | |  |  | | |  | | |  | |  | |
| 1) Never | 12.0 | 9.0 | 18.0 | 735 |  | 14.0 | 10.0 | 23.0 | 1815 |  | 16.0 | 12.0 | 27.0 | 437 |  | 21.0 | 14.0 | 33.0 | 621 |  | | 25.0 | | 16.0 | | 46.0 | | 295 | |  | 36.5 | | | 21.0 | | | 77.0 | | 218 | |
| 2) <20 cpd | 11.0 | 9.0 | 16.0 | 259 |  | 14.0 | 10.0 | 21.0 | 1263 |  | 15.0 | 10.0 | 24.0 | 344 |  | 20.0 | 12.0 | 37.0 | 591 |  | | 27.0 | | 16.0 | | 55.0 | | 242 | |  | 35.0 | | | 19.0 | | | 71.5 | | 224 | |
| 3) 20 cpd | 12.0 | 9.0 | 17.0 | 369 |  | 14.5 | 10.0 | 23.0 | 1382 |  | 16.0 | 11.0 | 27.0 | 365 |  | 21.0 | 13.0 | 37.0 | 748 |  | | 29.0 | | 16.0 | | 52.0 | | 409 | |  | 35.5 | | | 21.0 | | | 68.0 | | 416 | |
| 4) >20 cpd | 13.0 | 9.0 | 19.0 | 162 |  | 16.0 | 11.0 | 26.0 | 608 |  | 17.0 | 12.0 | 26.0 | 147 |  | 21.0 | 13.0 | 40.0 | 377 |  | | 28.0 | | 18.0 | | 52.0 | | 246 | |  | 42.0 | | | 23.0 | | | 84.0 | | 382 | |
| 5) Formerly | 13.0 | 11.0 | 20.0 | 237 |  | 17.0 | 12.0 | 27.0 | 994 |  | 18.0 | 12.0 | 28.0 | 276 |  | 25.0 | 15.0 | 44.0 | 578 |  | | 31.0 | | 18.0 | | 59.0 | | 263 | |  | 40.0 | | | 22.0 | | | 72.0 | | 278 | |
|  | Γ=0.009 | p=0.73 |  |  |  | Γ=0.034 | p=0.008 |  |  |  | Γ=0.021 | p=0.39 |  |  |  | Γ=0.017 | p=0.36 |  |  |  | | Γ=0.041 | | p=0.10 | |  | |  | |  | Γ=0.049 | | | p=0.061 | | |  | |  | |
| **AST** |  |  |  |  |  |  |  |  |  |  |  |  |  |  |  |  |  |  |  |  | |  | |  | |  | |  | |  |  | | |  | | |  | |  | |
| 1) Never | 10.0 | 9.0 | 12.0 | 735 |  | 11.0 | 9.0 | 13.0 | 1815 |  | 10.0 | 9.0 | 13.0 | 437 |  | 11.0 | 9.0 | 14.0 | 621 |  | | 12.0 | | 10.0 | | 16.0 | | 295 | |  | 14.0 | | | 11.0 | | | 21.0 | | 218 | |
| 2) <20 cpd | 10.0 | 8.0 | 12.0 | 259 |  | 10.0 | 9.0 | 12.0 | 1263 |  | 10.0 | 9.0 | 13.0 | 344 |  | 11.0 | 9.0 | 14.0 | 591 |  | | 13.0 | | 10.0 | | 17.0 | | 242 | |  | 14.0 | | | 11.0 | | | 21.0 | | 224 | |
| 3) 20 cpd | 10.0 | 8.0 | 11.0 | 369 |  | 10.0 | 9.0 | 12.0 | 1382 |  | 10.0 | 9.0 | 13.0 | 365 |  | 11.0 | 9.0 | 15.0 | 748 |  | | 13.0 | | 10.0 | | 17.0 | | 409 | |  | 14.0 | | | 11.0 | | | 21.0 | | 416 | |
| 4) >20 cpd | 9.0 | 8.0 | 11.0 | 162 |  | 10.0 | 9.0 | 13.0 | 608 |  | 11.0 | 9.0 | 13.0 | 147 |  | 11.0 | 9.0 | 14.0 | 377 |  | | 13.0 | | 11.0 | | 18.0 | | 246 | |  | 15.0 | | | 12.0 | | | 25.0 | | 382 | |
| 5) Formerly | 10.0 | 8.0 | 12.0 | 237 |  | 11.0 | 9.0 | 14.0 | 994 |  | 10.0 | 9.0 | 13.0 | 276 |  | 12.0 | 10.0 | 15.0 | 578 |  | | 13.0 | | 10.0 | | 17.0 | | 263 | |  | 14.0 | | | 11.0 | | | 20.0 | | 278 | |
|  | Γ=-0.166 | p<0.0001 |  |  |  | Γ=-0.038 | p=0.006 |  |  |  | Γ=0.006 | p=0.83 |  |  |  | Γ=0.033 | p=0.087 |  |  |  | | Γ=0.077 | | p=0.003 | |  | |  | |  | Γ=0.095 | | | p=0.0003 | | |  | |  | |
| **ALT** |  |  |  |  |  |  |  |  |  |  |  |  |  |  |  |  |  |  |  |  | |  | |  | |  | |  | |  |  | | |  | | |  | |  | |
| 1) Never | 13.0 | 10.0 | 18.0 | 735 |  | 13.0 | 10.0 | 19.0 | 1815 |  | 14.0 | 11.0 | 20.0 | 437 |  | 15.0 | 11.0 | 21.0 | 621 |  | | 16.0 | | 13.0 | | 23.0 | | 295 | |  | 19.0 | | | 14.0 | | | 28.0 | | 218 | |
| 2) <20 cpd | 12.0 | 9.0 | 17.0 | 259 |  | 12.0 | 9.0 | 18.0 | 1263 |  | 13.0 | 10.0 | 18.0 | 344 |  | 14.0 | 11.0 | 21.0 | 591 |  | | 17.0 | | 13.0 | | 25.0 | | 242 | |  | 17.0 | | | 13.0 | | | 25.5 | | 224 | |
| 3) 20 cpd | 12.0 | 9.0 | 16.0 | 369 |  | 13.0 | 10.0 | 17.0 | 1382 |  | 13.0 | 10.0 | 18.0 | 365 |  | 14.0 | 10.0 | 20.0 | 748 |  | | 15.0 | | 11.0 | | 22.0 | | 409 | |  | 17.0 | | | 12.0 | | | 26.0 | | 416 | |
| 4) >20 cpd | 11.0 | 8.0 | 15.0 | 162 |  | 13.0 | 10.0 | 19.0 | 608 |  | 13.0 | 10.0 | 18.0 | 147 |  | 15.0 | 11.0 | 21.0 | 377 |  | | 17.0 | | 12.0 | | 25.0 | | 246 | |  | 18.0 | | | 12.0 | | | 30.0 | | 382 | |
| 5) Formerly | 14.0 | 11.0 | 19.0 | 237 |  | 15.0 | 11.0 | 21.0 | 994 |  | 14.0 | 11.0 | 20.5 | 276 |  | 17.0 | 12.0 | 24.0 | 578 |  | | 18.0 | | 12.0 | | 25.0 | | 263 | |  | 21.0 | | | 14.0 | | | 30.0 | | 278 | |
|  | Γ=-0.135 | p<0.0001 |  |  |  | Γ=-0.036 | p=0.006 |  |  |  | Γ=-0.056 | p=0.028 |  |  |  | Γ=-0.035 | p=0.058 |  |  |  | | Γ=-0.030 | | p=0.24 | |  | |  | |  | Γ=-0.008 | | | p=0.77 | | |  | |  | |
|  |  |  |  |  |  |  |  |  |  |  |  |  |  |  |  |  |  |  |  |  | |  | |  | |  | |  | |  |  | | |  | | |  | |  | |

Note. For the quantification of the Gamma rank correlations (Γ) and the corresponding p-values, former smokers were excluded.
