# Supplementary material for: Liver Enzymes: Interaction Analysis of Smoking with Alcohol Consumption or BMI, Comparing AST and ALT to γ-GT
Source: PLoS One. 2011 Nov 22;6(11):e27951. doi: 10.1371/journal.pone.0027951 (PMC3222662; doi:10.1371/journal.pone.0027951)
Supplement: Table S2 — Liver enzyme activities according to smoking * body mass index strata. (DOC) [file pone.0027951.s002.doc]

| **Table S2.** Median activities (interquartile range) of liver enzymes according to smoking * body mass index strata. | | | | | | | | | | | | | | | |
| --- | --- | --- | --- | --- | --- | --- | --- | --- | --- | --- | --- | --- | --- | --- | --- |
|  |  |  |  |  |  |  |  |  |  |  |  |  | |  |  |
|  |  |  |  |  |  |  |  |  |  |  |  |  | |  |  |
| **smoking** | **BMI < 25 kg/m²** | | |  |  | **BMI 25 to < 30 kg/m²** | | | |  | **BMI >= 30 kg/m²** | | | |  |
|  | median | IQR | | n |  | median | IQR | | n |  | median | | IQR | | n |
|  |  |  |  |  |  |  |  |  |  |  |  | |  |  |  |
| **γ-GT** |  |  |  |  |  |  |  |  |  |  |  | |  |  |  |
| 1) Never | 12.0 | 9.0 | 18.0 | 1420 |  | 18.0 | 12.0 | 29.0 | 2080 |  | 24.0 | | 16.0 | 40.0 | 621 |
| 2) <20 cpd | 12.0 | 9.0 | 19.0 | 1460 |  | 19.0 | 13.0 | 35.0 | 1119 |  | 27.5 | | 16.0 | 50.0 | 344 |
| 3) 20 cpd | 15.0 | 10.0 | 25.0 | 1848 |  | 21.0 | 13.0 | 35.0 | 1472 |  | 25.0 | | 17.0 | 43.0 | 369 |
| 4) >20 cpd | 17.0 | 11.0 | 34.0 | 869 |  | 23.0 | 14.0 | 41.0 | 786 |  | 27.0 | | 17.0 | 50.0 | 267 |
| 5) Formerly | 14.0 | 10.0 | 22.0 | 635 |  | 21.0 | 14.0 | 37.0 | 1442 |  | 29.0 | | 18.0 | 50.0 | 549 |
|  | Γ=0.185 | p<0.0001 |  |  |  | Γ=0.106 | p<0.0001 |  |  |  | Γ=0.058 | | p=0.009 |  |  |
| **AST** |  |  |  |  |  |  |  |  |  |  |  | |  |  |  |
| 1) Never | 10.0 | 9.0 | 12.0 | 1420 |  | 11.0 | 9.0 | 13.0 | 2080 |  | 12.0 | | 10.0 | 15.0 | 621 |
| 2) <20 cpd | 10.0 | 9.0 | 13.0 | 1460 |  | 11.0 | 9.0 | 14.0 | 1119 |  | 12.0 | | 10.0 | 15.0 | 344 |
| 3) 20 cpd | 11.0 | 9.0 | 14.0 | 1848 |  | 11.0 | 9.0 | 14.0 | 1472 |  | 12.0 | | 9.0 | 14.0 | 369 |
| 4) >20 cpd | 11.0 | 9.0 | 15.0 | 869 |  | 11.0 | 9.0 | 14.0 | 786 |  | 11.0 | | 10.0 | 15.0 | 267 |
| 5) Formerly | 11.0 | 9.0 | 13.0 | 635 |  | 11.0 | 9.0 | 14.0 | 1442 |  | 12.0 | | 10.0 | 16.0 | 549 |
|  | Γ=0.103 | p<0.0001 |  |  |  | Γ=0.040 | p=0.002 |  |  |  | Γ=-0.031 | | p=0.20 |  |  |
| **ALT** |  |  |  |  |  |  |  |  |  |  |  | |  |  |  |
| 1) Never | 12.0 | 9.0 | 16.0 | 1420 |  | 15.0 | 11.5 | 21.0 | 2080 |  | 19.0 | | 14.0 | 27.0 | 621 |
| 2) <20 cpd | 11.0 | 9.0 | 16.0 | 1460 |  | 16.0 | 11.0 | 22.0 | 1119 |  | 19.0 | | 14.0 | 27.0 | 344 |
| 3) 20 cpd | 12.0 | 9.0 | 17.0 | 1848 |  | 15.0 | 11.0 | 21.0 | 1472 |  | 18.0 | | 14.0 | 26.0 | 369 |
| 4) >20 cpd | 12.0 | 9.0 | 18.0 | 869 |  | 16.0 | 11.0 | 23.0 | 786 |  | 19.0 | | 14.0 | 27.0 | 267 |
| 5) Formerly | 13.0 | 10.0 | 17.0 | 635 |  | 16.0 | 12.0 | 22.0 | 1442 |  | 20.0 | | 15.0 | 28.0 | 549 |
|  | Γ=0.039 | p=0.002 |  |  |  | Γ=0.015 | p=0.23 |  |  |  | Γ=-0.009 | | p=0.70 |  |  |
|  |  |  |  |  |  |  |  |  |  |  |  | |  |  |  |

Note. For the quantification of the Gamma rank correlations (Γ) and the corresponding p-values, former smokers were excluded.
